# Time to do something about reproducibility

**DOI:** 10.7554/eLife.03981

**Published:** 2014-12-10

**Authors:** Sean J Morrison

**Affiliations:** **Sean J Morrison** is an Investigator of the Howard Hughes Medical Institute, and Director of the Children’s Research Institute at the University of Texas Southwestern Medical Center, Dallas, USA. He is also the Mary McDermott Cook Chair in Pediatric Genetics and Director of the Hamon Laboratory for Stem Cells and Cancer at UTSW, and a Cancer Prevention and Research Institute of Texas Scholarsean.morrison@utsouthwestern.edu

**Keywords:** Reproducibility Project: Cancer Biology, methodology, open science, reproducibility, replication, blood stem cell

## Abstract

Individual scientists, scientific communities and scientific journals can do more to assess the publication of irreproducible results, to promote good science, and to increase the efficiency with which the scientific community self-corrects.

Irreproducible studies that side-track fields, waste resources and impede progress are a common frustration in academic science. Several analyses of certain subsets of cancer studies have concluded that most were not reproducible (Ioannidis et al., 2009; Prinz et al., 2011; Begley and Ellis, 2012). However, others have argued there are bound to be errors, but that science is self-correcting and that the system is working (Bissell, 2013). Where one stands on the matter depends partly on the size of the problem. In an effort to estimate how big the problem is, *eLife* has agreed to be the publisher for a project that will systematically assess the fraction of high-impact cancer studies whose major results can readily be reproduced.

This project—called the Reproducibility Project: Cancer Biology—has used a set of defined metrics to objectively identify 50 of the highest impact cancer studies, published between 2010 and 2012, that described observations that could be independently tested (Errington et al., 2014). The papers were not selected based on any controversy or suspicion that they are, or are not, reproducible. Members of the Reproducibility Project are in the process of designing experiments, which will be reviewed and approved in advance, to independently determine what percentage of these studies can be reproduced (see Box 1).

Box 1. **Details of the Reproducibility Project: Cancer Biology**

The Reproducibility Project: Cancer Biology is a collaboration between the Center for Open Science (a non-profit foundation dedicated to promoting openness, integrity, and reproducibility in scientific research) and the Science Exchange (a network of laboratories that performs assays on a fee-for-service basis, often in core facilities at academic institutions or in contract research organizations).

The Reproducibility Project is using a Registered Report/Replication Study approach to publish its work and results. The team replicating the study first submits a Registered Report that explains how it intends to replicate selected experiments from the original paper. The corresponding author of the original paper is contacted to suggest potential referees, to identify referees who should be excluded and, if they wish, to submit a review of the Registered Report.

Each Registered Report will be peer reviewed by several experts, including a statistician. Once the reviews have been received, a Reviewing Editor oversees a consultation between the referees and a decision letter listing essential revisions is sent to the authors of the report. The author of the original paper is not involved in the consultation process, but the Reviewing Editor can decide to consult him/her on specific points.

Once the Registered Report has been revised satisfactorily, it will be published. The replication team will then start to replicate the experiments, following the protocols detailed in the Registered Report: irrespective of the outcome, the results will be published as a Replication Study after peer review to check that the experiments were carried out in accordance with the protocols contained in the Registered Report.

To be clear, there is no reason to believe that the reproducibility problem is any more acute in cancer research than in other fields. The issue has just gotten more attention in the field of cancer biology, due partly to efforts to translate results into new therapies.

The Reproducibility Project itself is an experiment, and it remains to be seen whether this is an effective way of assessing the reproducibility of academic science. In principle, the findings of the Reproducibility Project could be undermined by the same sources of error it is attempting to address. One obvious concern is whether the laboratories that perform the replication studies on behalf of the Reproducibility Project have the expertise, experience and determination to successfully repeat the sometimes complex experiments described in the studies they examine. The Reproducibility Project has considered these issues and has promised to address them openly. It may not be perfect, but it is a credible effort to address an important question. Only time will tell whether the Reproducibility Project gets it right and whether its conclusions are ultimately sustained by independent studies.

The findings that emerge from the Reproducibility Project will often defy binary categorization into right and wrong. The project is not designed to assess the reproducibility of all aspects of the selected studies, only a subset of key experiments in each paper. This means that sometimes the replication attempt will not be comprehensive enough to draw any global conclusion about the replicability of a given study as a whole, instead focussing on the replicability of certain findings within the study. Consequently, this means that we may not be able to draw any conclusion about the major findings in some cases.

The findings that emerge from the Reproducibility Project will often defy binary categorization into right and wrong.

Considering the cancer biology literature as a whole, some studies may be completely right and some may be completely wrong. But there are likely to be many in the middle, with some reproducible findings that move the field forward, as well as other results that are not reproducible. The ultimate goal in science is to arrive at the truth, not to assign blame. Thus, our greatest hope is that authors will work with the Reproducibility Project and with their colleagues to figure out together what is reproducible so cancer biology can move forward on a sound footing and to efficiently translate results to benefit patients.

Irreproducible results can arise in many different ways. At one end of the spectrum are careful and well-meaning scientists who arrive at an incorrect interpretation as a result of an undetected technical problem that nobody could have foreseen—such as a reagent that does not work as expected. As long as the laboratory cooperates with efforts to get to the bottom of the problem and to correct the scientific record, this is not bad science. This is how a self-correcting system should work. At the other end of the spectrum lie laboratories who don't let controls or contradictory data get in the way of a good story and who do not play a constructive role in correcting the scientific record when their data turn out to be irreproducible or incorrectly interpreted. This is bad science. While the aim of the Reproducibility Project is not to determine why results are irreproducible, information on the fraction of key experiments that cannot be reproduced will provide data for introspection within the scientific community.

Self-correction is a comforting idea but can be a painfully inefficient process. And there is a legitimate question about whether the self-correcting character of science is efficient enough to provide an appropriate return on the public's investment in science (Collins and Tabak, 2014). We can all think of research areas that were launched by high-profile papers with revolutionary ideas that were not carefully tested. In my own field of stem cell biology a series of high-profile studies around the year 2000 claimed that blood-forming and other stem cells transdifferentiate into cells belonging to developmentally unrelated tissues under physiological conditions. This led to an explosion of hundreds of studies that all claimed to observe transdifferentiation among tissues in a way that threatened our understanding of developmental lineage relationships and the regulation of fate determination. However, most of these studies turned out not to be reproducible (Wagers et al., 2002; Balsam et al., 2004), and the rare events that were reproduced were explained by cell fusion rather than transdifferentiation (Alvarez-Dolado et al., 2003; Vassilopoulos et al., 2003; Wang et al., 2003). This episode illustrated how the power of suggestion could cause many scientists to see things in their experiments that weren't really there and how it takes years for a field to self-correct.

The transdifferentiation episode is not an isolated example. Studies with revolutionary ideas commonly lead to many follow-on studies that build on the original message without ever rigorously testing the central ideas. Under these circumstances dogma can arise like a house of cards, all to come crumbling down later when somebody has the energy to do the careful experiments and the courage to publish the results.

Cancer research has a remarkable track record of yielding discoveries that illuminate the biology of cancer and lead to new therapies that save and extend lives. But to be responsible stewards of the public's investment in this work we have to maximize the pace of discovery and the efficiency with which discoveries get translated to the benefit of patients. By gauging the fraction of high-impact results that are not reproducible, we can consider what further steps should be taken to promote good science.

Individual scientists, the fields in which we collectively work, and the journals that publish our results, all have the potential to do more to promote good science. One key distinction between good science, marked by effective self-correction, and myth-building is the extent to which scientists follow the scientific method. This scientific method is fundamental and yet is not always followed by scientists. Many scientists, like most humans, base their opinions and conclusions more on intuition than on careful experimentation and ignore the data that contradict intuitively attractive models. This is a major source of irreproducible results and of ideas that launch a thousand ships in the wrong direction. It is time to redouble our efforts to explicitly emphasize the scientific method when training graduate students, postdocs and junior faculty (Collins and Tabak, 2014). It's not science unless conclusions are rigorously tested and consistent with the data.

Scientific societies can do more to foster good science and to emphasize efficient self-correction rather than just being political organizations that promote their members. Big ideas can be stimulating, but if they are not right they are a setback. Some laboratories publish one irreproducible study after another in high-impact journals, collecting data to support their intuition, and paying little attention to whether or not the data truly support the conclusions. Anybody can make a mistake, but labs that repeatedly publish irreproducible results, and fail to engage with colleagues who are trying to resolve the inconsistencies, hold fields back. Scientific societies should take this into account when inviting speakers at annual meetings and candidates for leadership positions.

Too often journals publish papers and then do nothing when the papers turn out to be fatally flawed. Every journal should insist on a correction mechanism that is triggered when there is compelling reason to believe the original results are either not reproducible or misinterpreted. In an ideal world the original authors would correct the record, clarifying the original conclusions that still stand, as well as those they now interpret differently. In practice, this usually does not happen, even in the case of studies that are widely acknowledged in private conversations to be wrong.

One of the goals of eLife is to introduce innovations that have the potential to enhance our stewardship of the scientific record. Publication of the papers produced by the Reproducibility Project is one such experiment. Allowing reviewers to know each others' identities, and to discuss their reviews before returning comments to the authors, is another. Allowing authors to publish significant updates to papers in the journal is a third innovation that provides an opportunity for authors to publish important corrections, modifications or reinterpretations in light of significant new data. The editors of *eLife* will continue to look for appropriate ways to enhance the efficiency with which good science is published and bad science is corrected. In the meantime, measuring the magnitude of the problem with efforts like the Reproducibility Project: Cancer Biology is an important step in the right direction.
